# EEG-based detection of early functional brain changes in subjective cognitive decline: a prospective cohort study

**DOI:** 10.1186/s13195-025-01875-8

**Published:** 2025-12-29

**Authors:** Nayoung Ryoo, Ji Yong Park, Chunghwee Lee, SeongHee Ho, Yun Jeong Hong, Jee Hyang Jeong, Kee Hyung Park, Min Jeong Wang, Seong Hye Choi, SangYun Kim, Young Chul Youn, Euijin Kim, Sungkean Kim, Dong Won Yang

**Affiliations:** 1 Department of Neurology, Eunpyeong St. Mary’s Hospital, The Catholic University of Korea, Seoul, South Korea; 2 Department of Applied Artificial Intelligence, Hanyang University, Ansan, South Korea; 3 Department of Neurology, Seoul St. Mary’s Hospital, The Catholic University of Korea, Seoul, South Korea; 4 Department of Neurology, Uijeongbu St. Mary’s Hospital, The Catholic University of Korea, Uijeongbu City, South Korea; 5 Department of Neurology, Ewha Womans University Seoul Hospital, Ewha Womans University School of Medicine, Seoul, South Korea; 6 Department of Neurology, Gachon University Gil Medical Center, Incheon, South Korea; 7 Department of Neurology, Roa Neurology Clinic, Seongnam, South Korea; 8 Department of Neurology, Inha University College of Medicine, Incheon, South Korea; 9 Department of Neurology, Seoul National University College of Medicine and Seoul National University Bundang Hospital, Seongnam, South Korea; 10 Department of Neurology, Chung-Ang University College of Medicine, Seoul, South Korea; 11 Department of Human-Computer Interaction, Hanyang Univercity, Ansan, South Korea

**Keywords:** Subjective cognitive decline, Electroencephalography, Amyloid PET, Machine learning, Alzheimer's disease, Early diagnosis, Biomarkers

## Abstract

**Background:**

Subjective cognitive decline (SCD) has been recognized as a preclinical stage of Alzheimer’s disease. However, the identification of early functional brain changes remains challenging. This study investigated the functional brain changes in SCD using longitudinal EEG and evaluate the feasibility of EEG features as scalable biomarkers for identifying amyloid burden and cognitive decline using an interpretable machine learning framework.

**Methods:**

We analyzed 120 individuals with SCD enrolled in a multicenter prospective cohort (the CoSCo study) at baseline and after a 2-year follow-up. Participants were classified as amyloid-positive (A + SCD) or amyloid-negative (A − SCD). Spectral power and graph theory-based network analyses were conducted. Also, we trained machine learning classifiers to distinguish between the groups and interpreted the predictions of classifiers using SHAP.

**Results:**

At both baseline and follow-up, the A + SCD group exhibited elevated low-frequency (delta and theta) activity and reduced alpha activity compared to the A − SCD group. The EEG-based classifiers distinguished A + SCD from A-SCD individuals with high performance, outperforming a classifier based on demographic data. The results of SHAP analysis confirmed the importance and relative contribution of selected EEG features.

**Conclusions:**

Longitudinal EEG, when combined with interpretable machine learning, can detect and track the functional alterations of brain related to amyloid pathology in preclinical AD. Our findings support the feasibility of EEG as a non-invasive, scalable, and sensitive biomarker for risk stratification, before overt cognitive impairment emerges.

**Trial registration:**

This study was registered at the Clinical Research Information Service (CRIS) (cris.nih.go.kr/cris; # KCT0003397, Registration Date: December 21, 2018).

**Supplementary Information:**

The online version contains supplementary material available at 10.1186/s13195-025-01875-8.

## Background

Subjective cognitive decline (SCD) refers to a self-perceived decline in cognitive ability despite normal performance on standardized neuropsychological assessments. Over the past decade, SCD has emerged as a potential preclinical stage of Alzheimer’s disease (AD), offering a critical window for early intervention and prevention [1, 2]. Longitudinal studies have consistently shown that individuals with SCD are at an increased risk of progression to mild cognitive impairment (MCI) and dementia, including AD, compared to cognitively unimpaired individuals without subjective complaints [3].

Despite its clinical relevance, the pathological heterogeneity of SCD poses challenges for its early detection and stratification. Current biomarker modalities such as amyloid positron emission tomography (PET), cerebrospinal fluid (CSF) analysis, and magnetic resonance imaging (MRI) have significantly contributed to our understanding of the AD continuum [4, 5]. However, these techniques are often limited by their invasiveness, high cost, and limited accessibility, particularly for longitudinal monitoring in routine clinical settings [5].

Electroencephalography (EEG) has emerged as a promising alternative modality owing to its non-invasiveness, high temporal resolution, cost-effectiveness, and scalability [6]. EEG captures functional brain activity that may reflect early synaptic and network-level disruptions [7]. We employed spectral power analysis to test the ‘EEG slowing’ hypothesis, which links the synaptic dysfunction in AD continuum to a characteristic elevation in low-frequency (delta and theta) power [6, 7]. While this pattern is well-established in MCI and AD, there have been notable discrepancies across studies in SCD [8]. Also, there is a scarcity of longitudinal studies tracking how these spectral alterations change over time in SCD [8]. Furthermore, along with the ‘EEG slowing’ hypothesis, AD has been widely recognized as a ‘disconnection syndrome’, which indicates the pathology of AD targets brain networks disrupting the balance between local processing and global brain communication [9]. While spectral power analysis reflects isolated regional neural activity, graph theory-based network analysis with EEG can quantify these network-level functional alterations of brain as a global system. Although, several studies have investigated widespread network disintegration in MCI and AD [10], few studies have investigated the ‘disconnection syndrome’ with EEG in SCD. Therefore, it remains unclear whether the initial signs of network dysfunction of brain can also be detected in SCD and how these EEG phenomena evolve longitudinally at the preclinical SCD stage. Our study specifically addresses this gap by examining both spectral and network measures over a two-year follow-up in SCD individuals.

In this study, we aimed to investigate the functional brain changes in SCD using resting-state EEG measurements over a 2-year period. Specifically, we analyzed differences in spectral power and graph theory-based network activity between amyloid-positive (A + SCD) and amyloid-negative (A–SCD) individuals. Furthermore, we applied machine learning techniques to evaluate the utility of EEG-derived features for discriminating against amyloid status and assessed the potential of EEG as a non-invasive early biomarker for AD risk stratification.

## Methods

### Study design and participants

**Fig. 1:**
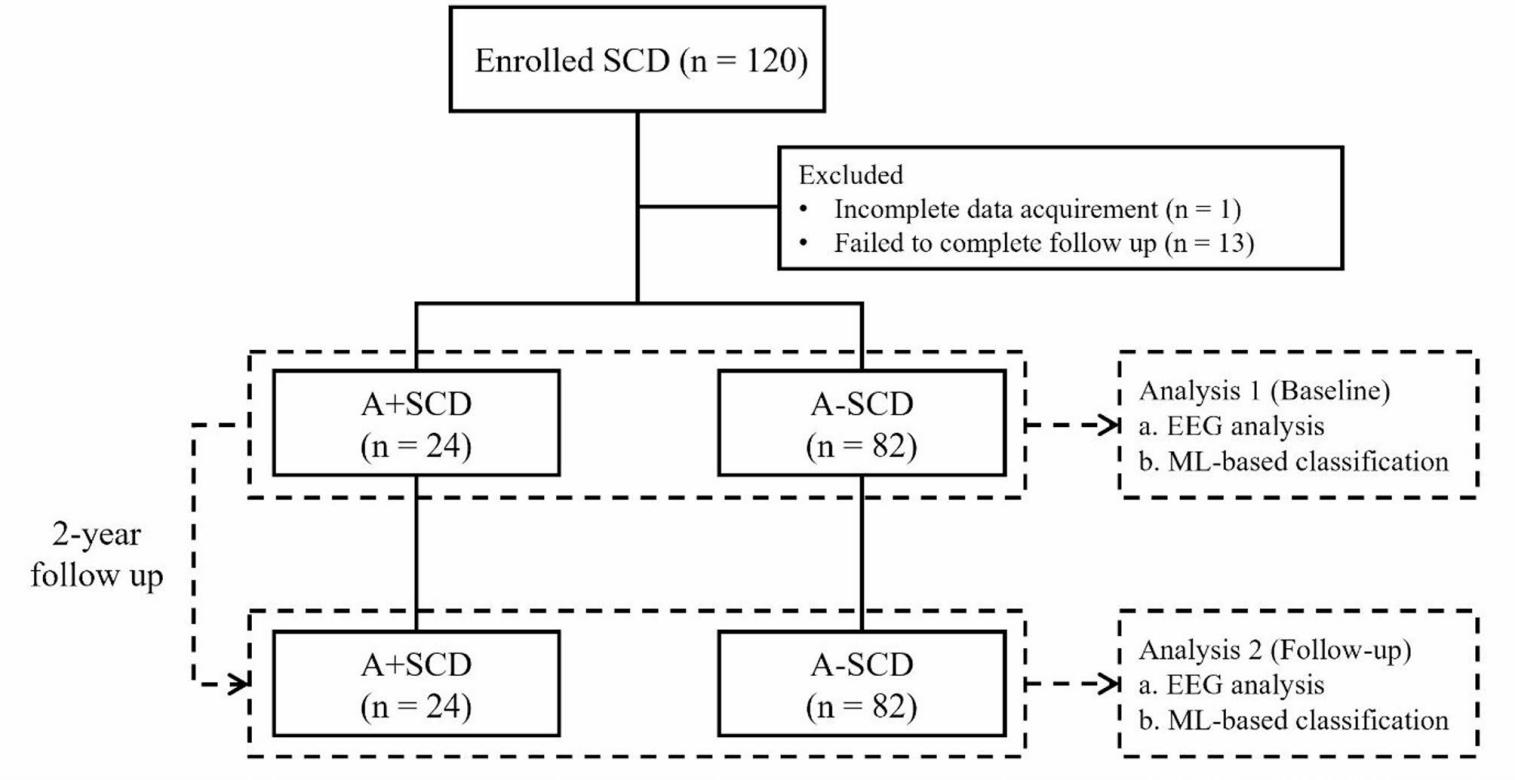
Flowchart of participant selection and study design Solid line, participant selection; dashed line, study design; SCD, subjective cognitive decline; A + SCD, amyloid-positive SCD; A–SCD, amyloid-negative SCD; ML, machine learning

This study was part of the Cohort Study to Identify Predictors for the Clinical Progression to Mild Cognitive Impairment or Dementia from Subjective Cognitive Decline (CoSCo), a multicenter prospective longitudinal study conducted across six academic medical centers in South Korea. Recruitment was conducted between November 2018 and December 2021. At baseline, the participants underwent demographic profiling, comprehensive neuropsychological testing, *apolipoprotein epsilon 4 (APOE4)* genotyping, structural brain MRI, amyloid PET imaging with ^18^F-florbetaben, and resting-state EEG. These assessments were repeated after 2 years of follow-up (Fig. 1). Detailed information regarding the study design has been described previously [11].

A total of 120 individuals aged >60 years with SCD were enrolled. All participants reported persistent cognitive complaints despite normal performance on standardized cognitive assessments, as determined by the Seoul Neuropsychological Screening Battery (SNSB) [12]. The inclusion criteria were a Clinical Dementia Rating (CDR) score of 0 and delayed recall scores on the Seoul Verbal Learning Test (SVLT) within the 7th to 50th percentiles adjusted for age, sex, and education [13]. The exclusion criteria were a history of neurological or psychiatric disorders, significant brain lesions, or abnormal laboratory results. Amyloid status was determined using ^18^F-florbetaben PET scans, and participants were classified into A + SCD and A − SCD groups based on brain amyloid plaque load scoring [14, 15].

### Neuropsychological assessments

Cognitive assessments included the Korean version of the Mini-Mental State Examination (K-MMSE), CDR, Digit Span Test–Forward (DST-F), Korean version of the Boston Naming Test (K-BNT), Rey Complex Figure Test–Copy (RCFT-C) and Delayed Recall (RCFT-DR), SVLT, Digit Symbol Coding (DSC), Controlled Oral Word Association Test (COWAT), Korean Trail Making Test B (K-TMT-B), and Korean Color Word Stroop Test (K-CWST) [12]. All test scores were normalized using large-scale Korean normative data (*n* = 1,100), with the 16th percentile used as the cutoff for cognitive impairment. The severity of subjective complaints was assessed using the Korean version of the Everyday Cognition questionnaire.

### Neuroimaging procedures

The MRI data were acquired using a 3.0 T scanner with three-dimensional T1-weighted fluid-attenuated inversion recovery, and susceptibility-weighted imaging sequences. White matter hyperintensities, lacunes, and cerebral microbleeds were visually assessed by a neurologist blinded to clinical data, using established rating criteria [13–16]. Experienced nuclear medicine physicians interpreted the PET images. Standardized uptake value ratios (SUVRs) were calculated using the cerebellar cortex as the reference region, and global SUVRs were derived from 28 AD-signature cortical regions. The PET and MRI images were co-registered and aligned using an automatic anatomical labeling atlas [15].

### EEG acquisition and processing

Resting-state EEG recordings were obtained using 19 scalp electrodes following the international 10–20 system. Participants were instructed to rest with their eyes closed for at least 3 min during the recording session. EEG recordings were conducted in an electromagnetically shielded and sound-attenuated room. EEG data were collected at baseline and follow-up assessments.

The EEG data were preprocessed using MATLAB R2021b (MathWorks, Natick, MA, USA) and the EEGLAB toolbox. Signals underwent bandpass filtering between 1 and 55 Hz, with additional artifact rejection applied through the CleanLine plugin, artifact subspace reconstruction, and independent component analysis based on ICLabel classification [17–19]. EEG data were segmented into 2-s epochs, and a random selection of 60 artifact-free epochs was retained for each participant.

The relative spectral power was estimated using Welch’s method with a Hamming window across five frequency bands: delta (1–4 Hz), theta (4–8 Hz), alpha (8–12 Hz), beta (12–30 Hz), and gamma (30–55 Hz). To evaluate functional brain connectivity, weighted phase-lag index matrices were constructed. Functional brain network metrics, including global and nodal clustering coefficient (CC), node strength (NS), and global efficiency (E), were extracted using the Brain Connectivity Toolbox [20]. CC refers to the tendency of nodes to cluster with its neighboring nodes, reflecting segregated clusters within the network, and NS indicates the strength of its connections with other nodes. Finally, E measures the efficiency of information exchange across the entire network, signifying its capacity for global integration [21]. Proportional thresholding ranging from 5% to 35% in 5% increments was applied to mitigate spurious connections [22]. Further details regarding the EEG preprocessing and analysis procedures are available in Supplementary Paragraph 1.

### Statistical analysis

Statistical analyses were conducted using SPSS 27 (SPSS, Inc., Chicago, IL, USA). The normality of distributions was verified using skewness and kurtosis thresholds (< 2 and < 7, respectively) [23]. Group differences in demographic and cognitive variables were assessed using independent t-tests and chi-square tests. In addition, paired t-tests were conducted to compare cognitive test scores between baseline and follow-up for both groups. Differences in EEG features were analyzed using analysis of covariance with age as a covariate. Effect sizes were reported as Cohen’s d and partial eta squared (η_p_^2^). Partial correlation analyses were conducted between EEG features and cognitive test scores, adjusting for age, and using bootstrapping with 5,000 iterations. Statistical significance was set at *P* < .05 (two-tailed).

### Machine learning classification

To evaluate the classification potential of the EEG features, we used an adaptive boosting (AdaBoost) classifier with sequential forward feature selection (a maximum of 20 features). The AdaBoost was selected for its effectiveness in handling class imbalance through its adaptive weighting mechanism and inherent robustness to overfitting in high-dimensional data [24, 25]. Models based on EEG features (spectral and network indices and age) were compared with those using demographic and cognitive data. All features were z-score-normalized. The model performance was assessed using stratified 10-fold cross-validation, reporting accuracy, sensitivity, specificity, Matthews correlation coefficient (MCC), and F1-score [26]. Hyperparameter tuning was not conducted to ensure generalization of the model and to avoid the risk of overfitting as much as possible. Permutation test with repetition of 5,000 (*P*_perm_) was adopted to assess whether the model performances were statistically significant, rather than being driven by chance. To mitigate the bias from class imbalance, Borderline Synthetic Minority Over-sampling Technique (Borderline-SMOTE) was used in the training data within each 10-fold cross-validation [27]. Additionally, Shapley Additive exPlanations (SHAP) analysis was adopted in models based on EEG features to evaluate their importance [28]. SHAP, a cooperative game-theory-based explanatory model, has been widely used to interpret artificial intelligence models. It assigns a SHAP value to each feature, representing the impact of the feature and its contribution to the model’s prediction. In this study, positive SHAP values indicated that the corresponding features contributed to the prediction of the A + SCD group.

### Ethics approval and consent to participate

This study was approved by the Institutional Review Board of Seoul St. Mary’s Hospital (IRB No. KC18ONDI0394, 2018-10-19). The study was conducted in accordance with the ethical standards of the Declaration of Helsinki and its later amendments or comparable ethical standards. Written informed consent was obtained from all participants.

## Results

### Demographic and clinical characteristics

Among the 120 participants, 25 were classified as A + SCD and 95 as A − SCD; 1 A + SCD was excluded because of incomplete data. Also, 13 A-SCD participants who failed to complete the study were excluded to ensure consistency between baseline and follow-up participants. During the 2-year follow-up, a total of 9 participants progressed to MCI or dementia: 5 participants from the A + SCD and 4 participants from A-SCD. These 9 participants were retained in all of our analyses. At baseline, no significant differences were observed between the groups in terms of sex distribution or educational level. However, participants in the A + SCD group were significantly older (*P* = .011) and had a higher proportion of *APOE4* carriers (*P* < .001). In contrast, the A − SCD group exhibited a significantly higher body mass index (*P* = .003), body fat percentage (*P* = .030), visceral fat (*P* = .001), and waist circumference (*P* = .004) (Table 1).

**Table 1:** Baseline demographic characteristics of the study participants

	A + SCD (*N* = 24)	A–SCD (*N* = 82)	*P*
Age (years)	73.42 ± 5.64	69.82 ± 6.05	**0.011**
Sex, male (n, %)	14 (58.3)	32 (39.0)	0.093
Education (years)	12.71 ± 4.07	11.04 ± 3.88	0.069
APOE4 carrier (n, %)	12 (50.0)	9 (11.0)	**< 0.001**
Global SUVR	1.64 ± 0.28	1.18 ± 0.09	**< 0.001**
BMI	23.14 ± 2.67	25.35 ± 3.20	**0.003**
Body fat (%)	26.10 ± 7.71	30.12 ± 7.90	**0.030**
Body muscle (%)	23.81 ± 5.01	24.52 ± 6.64	0.626
Visceral fat (%)	6.92 ± 2.77	9.77 ± 3.82	**0.001**
Waist circumference (cm)	81.61 ± 8.94	87.84 ± 9.16	**0.004**
Framingham cardiovascular risk score	10.98 ± 8.78	8.39 ± 7.06	0.139
K-ECOG	68.92 ± 20.92	70.68 ± 22.02	0.727
Periventricular WMH (grade 1/2/3)	18/3/3	53/17/12	0.792
Deep WMH (grade 1/2/3)	21/3/0	58/18/6	0.172
Lacune (n, %)	7 (8.5)	1 (4.2)	0.476
Cerebral microbleed (n, %)	3 (12.5)	5 (6.1)	0.296

Bold indicates a significant *P*-value (*P* < .05); A + SCD, amyloid-positive subjective cognitive decline; A–SCD, amyloid-negative subjective cognitive decline; APOE4, apolipoprotein epsilon 4; SUVR, standardized uptake value ratio; BMI, body mass index; K-ECOG, Korean version of Everyday Cognition; WMH, white-matter hyperintensity

Cognitive performance did not differ significantly between the two groups at baseline. However, at follow-up, the A + SCD group showed greater cognitive decline, with significantly lower scores on the K-MMSE (*P* = .001, *d* = 0.83), K-BNT (*P* = .022, *d* = 0.54), SVLT (*P* < .001, *d* = 0.86), and K-TMT-B (*P* < .001, *d* = 0.92) than the A − SCD group (Table 2).

**Table 2:** Cognitive tests of study participants at baseline and follow-up

	A + SCD (*N* = 24)	A–SCD (*N* = 82)	Cohen’s d (95% CI)	*P*
**Baseline**				
K-MMSE	26.88 ± 2.36	27.28 ± 1.85	0.20 (-0.25, 0.66)	0.379
DST-F	58.69 ± 33.12	63.25 ± 29.28	0.15 (-0.30, 0.61)	0.517
K-BNT	54.02 ± 30.71	59.71 ± 26.28	0.21 (-0.25, 0.66)	0.371
RCFT-C	60.83 ± 21.73	57.00 ± 22.24	-0.17 (-0.63, 0.28)	0.458
RCFT-DR	43.88 ± 26.44	47.79 ± 23.80	0.16 (-0.30, 0.62)	0.491
SVLT	23.13 ± 15.10	28.02 ± 13.06	0.36 (-0.10, 0.82)	0.122
DSC	52.82 ± 33.17	64.04 ± 24.06	0.43 (-0.03, 0.88)	0.070
COWAT	53.03 ± 31.62	52.98 ± 27.69	0.00 (-0.46, 0.45)	0.994
K-TMT-B	58.42 ± 22.86	63.86 ± 21.10	0.25 (-0.20, 0.71)	0.278
K-CWST	51.04 ± 29.87	56.97 ± 24.79	0.23 (-0.23, 0.68)	0.328
**Follow-up**				
K-MMSE	26.42 ± 2.78	28.10 ± 1.75	0.83 (0.36, 1.30)	**0.001**
DST-F	57.75 ± 32.73	69.63 ± 27.39	0.41 (-0.04, 0.87)	0.077
K-BNT	52.91 ± 36.56	68.32 ± 25.88	0.54 (0.08, 1.00)	**0.022**
RCFT-C	53.15 ± 24.22	52.37 ± 25.44	-0.03 (-0.49, 0.42)	0.894
RCFT-DR	48.67 ± 32.65	59.30 ± 27.40	0.37 (-0.09, 0.83)	0.113
SVLT	30.69 ± 26.00	52.21 ± 24.91	0.86 (0.38, 1.32)	**< 0.001**
DSC	57.83 ± 32.43	69.56 ± 24.89	0.44 (-0.02, 0.90)	0.061
COWAT	54.25 ± 28.11	57.11 ± 29.77	0.10 (-0.36, 0.55)	0.676
K-TMT-B	47.33 ± 25.90	67.73 ± 21.02	0.92 (0.45, 1.39)	**< 0.001**
K-CWST	50.96 ± 26.87	61.61 ± 28.79	0.38 (-0.08, 0.83)	0.109

Bold indicates a significant *P*-value (*P* < .05); A + SCD, amyloid-positive subjective cognitive decline; A–SCD, amyloid-negative subjective cognitive decline; CI, confidence interval; K-MMSE, Korean version of the Mini-Mental State Examination; DST-F, Digit Span Test: Forward; K-BNT, Korean version of the Boston Naming Test; RCFT-C, Rey Complex Figure Test–Copy; RCFT-DR, Rey Complex Figure Test–Delayed Recall; SVLT, Seoul Verbal Learning Test; DSC, Digit Symbol Coding; COWAT, Controlled Oral Word Association Test; K-TMT-B, Korean version of the Trail Making Test B; K-CWST, Korean version of the Color Word Stroop Test

Within-group comparisons showed several improvements in A-SCD. The K-MMSE (*P* = .001, *d* = -0.40), K-BNT (*P* < .001, *d* = -0.42), RCFT-DR (*P* < .001, *d* = -0.44), SVLT (*P* < .001, *d* = -1.09), and DSC (*P* = .012, *d* = -0.29) scores were significantly increased during the study period in A-SCD. In contrast, the A + SCD showed significant decrease in K-TMT-B (*P* = .020, *d* = 0.51) at follow-up compared to baseline (Table S1).

### EEG differences by amyloid status

EEG analyses at baseline revealed 31 features with significant differences between the A + SCD and A − SCD groups (Table 3). In the A + SCD group, the relative spectral power was significantly elevated in the delta (*n* = 10) and theta (*n* = 1) bands. Additionally, the CC (*n* = 3) and NS (*n* = 1) were higher in the delta band. In contrast, the A − SCD group demonstrated significantly greater relative spectral power in the alpha (*n* = 2) and beta (*n* = 1) bands, along with higher CC (*n* = 10) and NS (*n* = 3) in the alpha band. These results suggest increased low-frequency activity and reduced alpha network activity in the A + SCD group at baseline.

**Table 3:** EEG spectral and network features showing significant differences between the A + SCD and A–SCD groups at baseline

Frequency band	Feature	A + SCD (*N* = 24)	A–SCD (*N* = 82)	Effect size (Partial η^2^)	*P*
Delta	Fp1	34.42 ± 14.80	26.99 ± 12.14	0.07	**0.009**
	F7	41.49 ± 12.97	33.22 ± 14.23	0.05	**0.018**
	Fz	27.85 ± 13.93	21.84 ± 11.70	0.04	**0.031**
	F4	32.15 ± 15.19	25.11 ± 13.80	0.04	**0.048**
	T5	22.16 ± 12.45	16.80 ± 11.18	0.04	**0.048**
	Pz	28.95 ± 15.04	22.85 ± 10.40	0.04	**0.038**
	T6	23.31 ± 13.88	16.27 ± 10.99	0.06	**0.012**
	O1	26.39 ± 14.30	16.68 ± 11.82	0.10	**0.001**
	O2	24.41 ± 12.61	17.05 ± 12.66	0.05	**0.026**
	Global	28.99 ± 12.08	23.05 ± 10.16	0.05	**0.022**
	T3 (CC)	0.28 ± 0.03	0.26 ± 0.02	0.04	**0.050**
	C3 (CC)	0.28 ± 0.02	0.26 ± 0.02	0.05	**0.027**
	P3 (CC)	0.28 ± 0.02	0.27 ± 0.02	0.06	**0.011**
	T4 (NS)	4.80 ± 0.62	4.45 ± 0.74	0.04	**0.044**
Theta	O1	17.62 ± 10.27	12.24 ± 8.02	0.04	**0.044**
Alpha	O1	35.01 ± 19.74	48.02 ± 22.28	0.06	**0.015**
	O2	36.46 ± 20.77	49.24 ± 23.35	0.05	**0.025**
	Fp1 (CC)	0.28 ± 0.07	0.32 ± 0.09	0.04	**0.043**
	F7 (CC)	0.27 ± 0.06	0.31 ± 0.09	0.04	**0.043**
	F3 (CC)	0.27 ± 0.07	0.31 ± 0.09	0.04	**0.040**
	Fz (CC)	0.28 ± 0.07	0.32 ± 0.09	0.04	**0.043**
	F4 (CC)	0.28 ± 0.07	0.32 ± 0.09	0.04	**0.045**
	Cz (CC)	0.27 ± 0.06	0.32 ± 0.10	0.04	**0.032**
	C4 (CC)	0.27 ± 0.07	0.32 ± 0.09	0.05	**0.017**
	T5 (CC)	0.27 ± 0.07	0.31 ± 0.09	0.04	**0.050**
	Pz (CC)	0.28 ± 0.07	0.32 ± 0.10	0.04	**0.048**
	Global (CC)	0.27 ± 0.06	0.31 ± 0.09	0.04	**0.050**
	C4 (NS)	4.60 ± 1.96	5.63 ± 2.08	0.04	**0.035**
	Pz (NS)	4.93 ± 1.54	5.94 ± 2.23	0.04	**0.037**
	O2 (NS)	5.07 ± 1.40	5.98 ± 1.90	0.05	**0.026**
Beta	T3	18.89 ± 8.27	24.53 ± 9.73	0.04	**0.048**

### Bold indicates a significant P-value (P < .05); A + SCD, amyloid-positive subjective cognitive decline; A–SCD, amyloid-negative subjective cognitive decline; CC, clustering coefficient; NS, node strength

At follow-up, similar trends persisted, with 31 EEG features significantly different between the groups (Table 4). In the A + SCD group, the relative spectral power in the theta band markedly increased (*n* = 17). The A − SCD group exhibited higher network activity, particularly in the alpha (CC, *n* = 5; NS, *n* = 5) and gamma (CC, *n* = 2; NS, *n* = 2) bands. These findings indicated ongoing functional network degradation in the A + SCD group, particularly within the theta and alpha oscillatory domains.

**Table 4:** EEG spectral and network features showing significant differences between the A + SCD and A–SCD groups at 2-year follow-up

Frequency band	Feature	A + SCD (*N* = 24)	A–SCD (*N* = 82)	Effect size (Partial η^2^)	*P*
Theta	Fp1	17.19 ± 6.52	13.12 ± 6.74	0.05	**0.020**
	F3	19.92 ± 7.32	14.86 ± 8.47	0.06	**0.014**
	Fz	24.17 ± 9.30	17.66 ± 9.40	0.08	**0.004**
	F4	19.29 ± 7.70	14.86 ± 7.36	0.05	**0.020**
	T3	19.85 ± 10.23	14.81 ± 7.04	0.05	**0.017**
	C3	17.66 ± 7.26	13.62 ± 6.04	0.05	**0.020**
	Cz	19.19 ± 7.46	15.12 ± 6.01	0.06	**0.012**
	C4	17.64 ± 8.80	13.73 ± 6.36	0.04	**0.032**
	T4	18.74 ± 11.16	14.21 ± 6.42	0.05	**0.030**
	T5	20.20 ± 12.07	12.56 ± 7.16	0.07	**0.006**
	P3	17.96 ± 9.22	12.88 ± 6.18	0.06	**0.009**
	Pz	18.45 ± 8.38	13.70 ± 6.38	0.06	**0.010**
	P4	18.44 ± 10.00	12.72 ± 6.10	0.08	**0.003**
	T6	19.53 ± 11.54	12.99 ± 7.29	0.07	**0.005**
	O1	18.40 ± 9.81	12.08 ± 7.11	0.08	**0.004**
	O2	18.13 ± 10.93	11.87 ± 7.10	0.07	**0.005**
	Global	18.66 ± 7.82	13.83 ± 6.15	0.07	**0.006**
Alpha	F7 (CC)	0.27 ± 0.06	0.31 ± 0.08	0.04	**0.032**
	F3 (CC)	0.28 ± 0.06	0.31 ± 0.08	0.04	**0.037**
	Fz (CC)	0.28 ± 0.06	0.31 ± 0.08	0.04	**0.048**
	C3 (CC)	0.27 ± 0.06	0.31 ± 0.08	0.04	**0.036**
	P4 (CC)	0.28 ± 0.07	0.32 ± 0.08	0.04	**0.040**
	Fp1 (NS)	4.82 ± 1.36	5.61 ± 1.62	0.05	**0.027**
	Fp2 (NS)	4.72 ± 1.58	5.55 ± 1.65	0.04	**0.033**
	T5 (NS)	4.97 ± 1.21	5.67 ± 1.62	0.04	**0.040**
	P4 (NS)	5.12 ± 1.36	5.99 ± 1.93	0.04	**0.038**
	T6 (NS)	4.88 ± 1.35	5.67 ± 1.59	0.05	**0.022**
Gamma	Fp1 (CC)	0.10 ± 0.01	0.11 ± 0.01	0.04	**0.037**
	T6 (CC)	0.10 ± 0.01	0.11 ± 0.01	0.05	**0.016**
	Fz (NS)	1.75 ± 0.41	1.99 ± 0.39	0.05	**0.023**
	P4 (NS)	1.76 ± 0.25	1.91 ± 0.32	0.04	**0.048**

Bold indicates a significant *P*-value (*P* < .05); A + SCD, amyloid-positive subjective cognitive decline; A–SCD, amyloid-negative subjective cognitive decline; CC, clustering coefficient; NS, node strength

### Correlations between EEG and cognitive performance

Partial correlation analyses controlling for age were performed between EEG features and cognitive test scores (Tables S2 and S3). At baseline, the global SUVR was positively correlated with delta and theta activities and negatively correlated with alpha and beta activities. In the follow-up phase, similar relationships were observed, with theta activity showing positive correlations and gamma activity showing negative correlations with the SUVR (Fig. S1).

Delta and theta power were negatively correlated with performance on the K-MMSE, DST-F, K-BNT, SVLT, DSC, and K-CWST at baseline. In contrast, alpha power was positively correlated with K-MMSE, K-BNT, and COWAT scores. At follow-up, theta power remained negatively associated with the DST-F, K-BNT, DSC, COWAT, and K-TMT-B, whereas alpha and gamma power were positively correlated with the K-MMSE, K-BNT, RCFT-DR, COWAT, and K-CWST (Fig. S2).

### Classification performance and interpretation

Machine learning classification using EEG features demonstrated higher discriminative performance than demographic features in both phases (Table 5). At baseline, the EEG-based classifier achieved an accuracy of 0.917 (*P*_perm_ < 0.001), sensitivity of 0.867 (*P*_perm_ < 0.001), specificity of 0.929 (*P*_perm_ < 0.001), MCC of 0.784 (*P*_perm_ < 0.001), and F1-score of 0.821 (*P*_perm_ < 0.001) using 15 selected features. In contrast, the best-performing demographic-based classifier yielded a specificity of 0.964 (*P*_perm_ < 0.001) but had lower accuracy of 0.895 (*P*_perm_ < 0.001), sensitivity of 0.633 (*P*_perm_ < 0.001), MCC of 0.650 (*P*_perm_ < 0.001), and F1-score of 0.676 (*P*_perm_ < 0.001) using 9 features (Table S4).

**Table 5:** Best classification performance at baseline and follow-up

Case	Accuracy	Sensitivity	Specificity	MCC	F1-score
Baseline (EEG)	0.917 ± 0.076	0.867 ± 0.208	0.929 ± 0.076	0.784 ± 0.210	0.821 ± 0.175
Baseline (Demography)	0.895 ± 0.055	0.633 ± 0.296	0.964 ± 0.055	0.650 ± 0.263	0.676 ± 0.259
Follow-up (EEG)	0.907 ± 0.071	0.817 ± 0.229	0.928 ± 0.095	0.765 ± 0.166	0.794 ± 0.146
Follow-up (Demography)	0.850 ± 0.072	0.717 ± 0.334	0.892 ± 0.083	0.588 ± 0.281	0.643 ± 0.248

MCC, Matthews correlation coefficient; EEG, electroencephalography

At the follow-up, the EEG classifiers continued to outperform the demographic models. The EEG-based classifier achieved an accuracy of 0.907 (*P*_perm_ < 0.001), sensitivity of 0.817 (*P*_perm_ < 0.001), specificity of 0.928 (*P*_perm_ < 0.001), MCC of 0.765 (*P*_perm_ < 0.001), and F1-score of 0.794 (*P*_perm_ < 0.001) using 13 selected features. The demographic-based classifier achieved an accuracy of 0.850 (*P*_perm_ < 0.001), sensitivity of 0.717 (*P*_perm_ < 0.001), specificity of 0.892 (*P*_perm_ < 0.001), MCC of 0.588 (*P*_perm_ < 0.001), and F1-score of 0.643 (*P*_perm_ < 0.001) using 9 features (Table S5). Notably, age, although included in the EEG feature set, was not selected by the classifier in either phase, underscoring the independent discriminatory value of the EEG metrics.

**Fig. 2:**
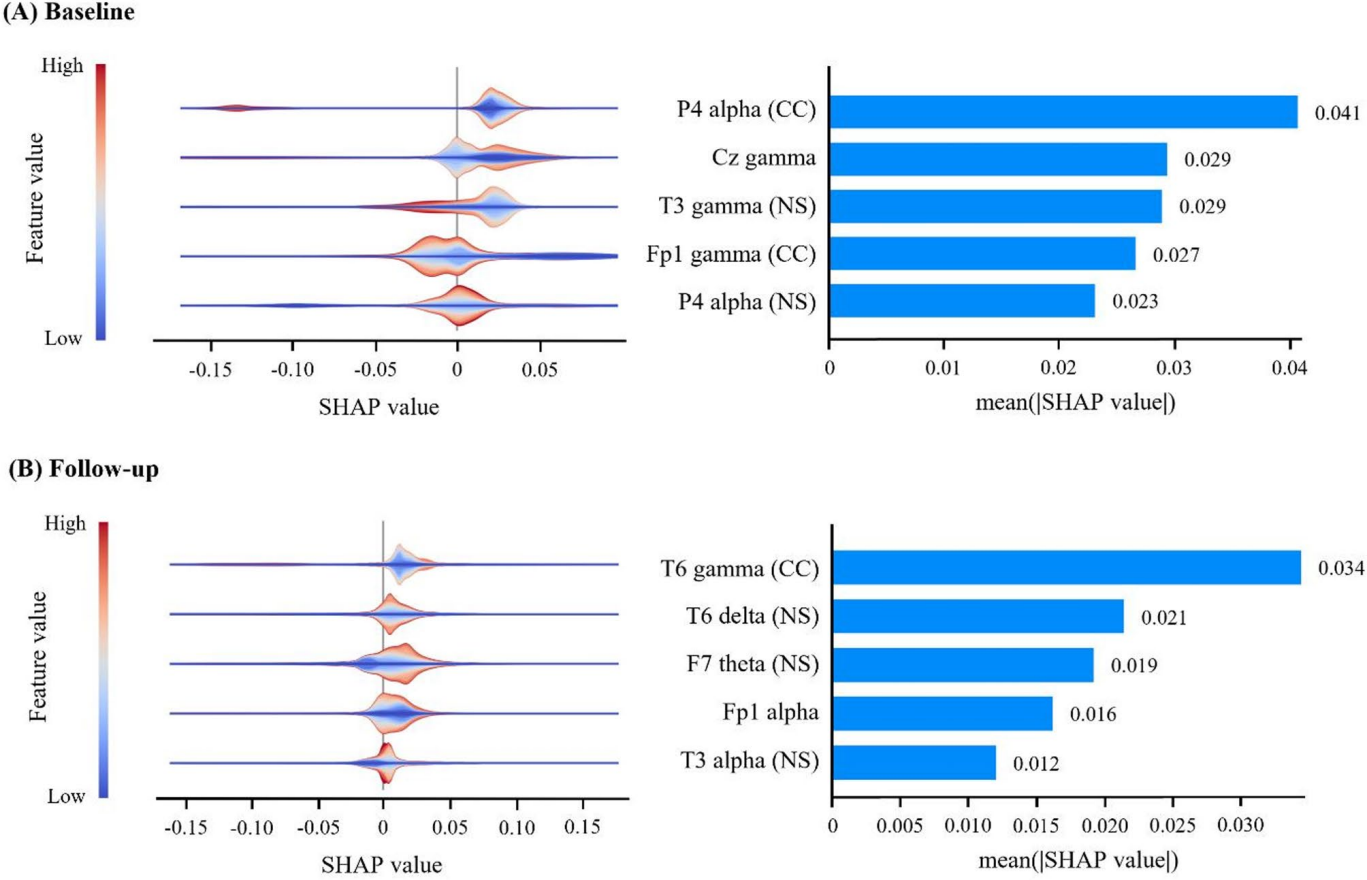
Violin and bar plots of SHAP in the top five features at (**A**) baseline and (**B**) follow-up SHAP, SHapley Additive exPlanations; CC, clustering coefficient; NS, node strength

To evaluate the importance and contribution of the features, SHAP values were investigated for models based on EEG features. Among selected features, top five features with the highest mean absolute SHAP values are shown (Fig. 2). At baseline, EEG features in alpha and gamma showed relatively higher SHAP values. Among the top five features, the alpha CC in P4 exhibited the highest mean absolute SHAP value of 0.041. The SHAP violin plot for the alpha CC in P4 was clustered on the positive side of the SHAP value, indicating that a lower alpha activity in P4 influenced the model’s prediction toward the A + SCD group (Fig. 2A). The feature with the highest mean absolute SHAP value was gamma CC in T6 at follow-up, with a statistically significant group difference (*P* = .016, η_p_^2^ = 0.05). The SHAP values of gamma CC in T6 were largely concentrated in the positive range, confirming that lower gamma activity pushed the model’s prediction toward the A + SCD group (Fig. 2B).

### EEG differences by APOE4 status

To investigate whether genetic risk of APOE4 influenced EEG characteristics in our study and to explore EEG differences according to APOE4 status, we additionally divided the participants into two groups: APOE4 carrier (*n* = 21), APOE4 non-carrier (*n* = 85). There was only significant difference in global SUVR (*P* < .001) between the groups at baseline (Table S6). The global SUVR was included as a covariate for further analysis. While cognitive performances were not different at baseline, K-MMSE (*P* = .038, d = 0.51) was shown to be higher in the APOE4 non-carrier (Table S7). In group comparisons of EEG features, the APOE4 carrier showed higher delta power (*n* = 1), alpha power (*n* = 1), beta network activity (CC, *n* = 5; NS, *n* = 2), and gamma power (*n* = 1), as well as lower theta power (*n* = 1) at baseline. In addition, at follow-up, theta power (*n* = 1), beta network (CC, *n* = 18; NS, *n* = 9; E, *n* = 1), and gamma network (NS, *n* = 2) were significantly higher in the APOE4 carrier, while delta power (*n* = 1), theta network (NS, *n* = 1), beta power (*n* = 1), and gamma power (*n* = 1) were decreased in the APOE4 carrier (Table S8).

## Discussion

### Main findings: longitudinal EEG alterations in SCD

In this study, we identified significant differences in EEG characteristics according to amyloid burden status in individuals with SCD. The A + SCD group exhibited increased low-frequency activities (delta and theta) and decreased alpha network activities at baseline and after 2 years of follow-up, despite no significant cognitive difference detected at baseline by standard neuropsychological testing. These findings suggest that EEG may detect early functional brain changes along the AD continuum that are not captured by traditional cognitive assessments.

This observation aligns with prior studies suggesting that functional neural changes, such as altered oscillatory dynamics, can precede clinically detectable cognitive impairments [7, 29]. EEG abnormalities, particularly in the low-frequency and alpha bands, have been reported in both amnestic MCI and SCD populations, indicating sensitivity to early synaptic disruption [30, 31]. Furthermore, standard neuropsychological assessments may lack sensitivity in capturing subtle cognitive decline in the preclinical stages of AD, underscoring the need for physiological biomarkers, such as EEG [32].

While these results support the general utility of EEG, a crucial test for any potential biomarker is its ability to translate group-level differences into reliable single-subject classification. Although several prior longitudinal and cross-sectional studies have investigated EEG alterations in SCD [8], they have primarily focused on group-level statistical trends. A key contribution of our study, therefore, is the demonstration of the feasibility of amyloid status classification using a machine learning framework. Furthermore, by employing SHAP, we could infer the neurophysiological drivers of the model’s predictions, adding a layer of interpretability. By showing that the multivariate patterns within an individual’s EEG can predict the amyloid status, our findings provide a stronger foundation for the use of EEG as a practical and scalable tool for early risk stratification. By detecting neural changes before the onset of clinical symptoms, EEG can facilitate timely intervention and stratification for clinical trials targeting the earliest stages of AD.

### Alterations in EEG activity

Our statistical results showed coherent patterns of increased delta activity at baseline and increased theta activity at follow-up. These results align with previous findings that low-frequency activities increase with amyloid accumulation and cognitive decline along the AD continuum [7, 33, 34]. Also, our analyses confirmed that the ‘EEG slowing’ phenomenon in the MCI and AD exists in preclinical SCD. Interestingly, the elevation of delta activity was detected even before objective cognitive decline became evident. This increment of delta at baseline might reflect the amyloid-induced synaptic dysfunction, which is the earliest change in the pathology of AD before cognitive decline occurs [35, 36]. Subsequently, the emergence of widespread theta increment may represent a progression of initial dysfunction. The temporal shift from delta to theta pattern of slowing over time could signify that the initial synaptic dysfunction and instability has evolved to disrupt attentional function, which is known to be linked to theta oscillations [37]. The significant difference in the K-TMT-B at follow-up, a test reliant on attentional control processes, also supports the interpretation of emergent theta activity.

In parallel with spectral changes, our results also provide clear evidence for the ‘disconnection syndrome’ hypothesis. The A + SCD exhibited reduced network activity in alpha at both baseline and follow-up. This reduced network pattern suggests that the segregation of the overall brain network, which is critical for cognitive processing, has been compromised persistently from the early of the disease process [36]. Notably, alpha oscillations have been known to be associated with memory function [38], and its reduction may indicate an early breakdown of cognitive systems.

The importance of longitudinal alterations in EEG patterns underscores the potential of EEG, notably delta and theta activity, as well as alpha network efficiency, as accessible, noninvasive biomarkers of early AD risk and amyloid-induced neural dysfunction. This suggests that EEG can capture the evolving functional impact of amyloid pathology, offering a scalable method for monitoring clinical progression before cognitive decline becomes evident.

Furthermore, we also investigated the EEG differences between the APOE4 carrier and APOE4 non-carrier to explore whether the APOE4 influenced EEG characteristics in our study and to examine EEG differences according to APOE4 status. Interestingly, the APOE4 carrier exhibited a distinct EEG pattern, most notably characterized by an overall increment in beta network activity. While these findings are preliminary, it is plausible that they reflect an early state of network hyperexcitability. The results might suggest that this increased beta network activity could be a manifestation of APOE4-driven neuroinflammatory processes altering the brain’s excitation-inhibition balance and leading aberrant neural firing [39]. In other words, this exploratory finding suggests possibility that beta networks are over-activated by APOE4-driven neuroinflammatory processes. To validate this hypothesis, regarding neuroinflammation and beta network, further study of preclinical stages of AD is warranted. Finally, the EEG pattern associated with APOE4 status was distinctly different from the patterns driven by amyloid burden, suggesting that these two AD risk factors impact the brain through at least partially independent mechanisms.

### SHAP-based interpretation of neural dysfunction

To move beyond group-level statistics and identify the feasibility of EEG features as biomarkers, the SHAP analysis was employed in the machine learning analysis (Fig. 2). At baseline, the most salient features were primarily related to the organization of high frequency networks (alpha and gamma) in frontal, temporal, and parietal hubs of the default mode network (DMN) [40]. Although regional alterations in scalp level might not be sufficient to explain source activity in specific brain areas, these consistent results with previous studies for abnormal DMN in cognitive decline might suggest that the earliest functional impact of amyloid may be a subtle destabilization of the brain’s circuits responsible for memory and attention [40].

After two years, the discriminative pattern evolved. While network alterations in high frequency remained, there was a notable emergence of disruptions in low-frequency networks. One plausible interpretation is that this emergence reflects a maladaptive compensatory response to the ongoing disruption of alpha network. As the efficient and long-range communication supported by the alpha network continues to degrade, the brain may attempt to compensate by over-recruiting slower oscillations. This pattern of increased low-frequency synchronization with reductions in high frequency connectivity has been previously reported in MCI and AD [41, 42]. It could lead to a state of pathological hypersynchrony, where local circuits become excessively synchronized but functionally isolated. Computational models support this hypothesis, linking amyloid-induced neural hyperactivity to both oscillatory slowing and excessive local clustering, which ultimately drives network fragmentation and a loss of global integration [34]. Therefore, the new emergence of low-frequency network features may represent the electrophysiological result of shift from initial impaired long-range communications to a more advanced state of widespread network breakdown.

A particularly interesting finding highlighted by our SHAP analysis was the early and persistent emergence of gamma network activity. While gamma alterations, such as increased power, have typically been reported in later stages like MCI or AD, often interpreted as a sign of compensatory hyperactivation [43], our results suggest their potential relevance even at the preclinical SCD. This observation raises the possibility that gamma dysfunction may not exclusively be a ‘late marker’ of the disease. Alternatively, we hypothesize that this early emergence of gamma could reflect the high vulnerability of GABAergic interneurons to early amyloid toxicity. As these interneurons are critical for generating and regulating gamma oscillations, their disruption could represent one of the initial neurophysiological consequences of amyloid pathology, ultimately leading to network hypersynchrony [44]. This hypothesis is conceptually supported by an animal study demonstrating a close and bidirectional relationship between amyloid pathology and gamma activity, where manipulating gamma oscillations has been shown to impact amyloid burden [45].

### Classification performances and clinical implications

We also evaluated the feasibility of EEG features in classifying the amyloid status at both baseline and follow-up using machine learning models (Table 5). To overcome the significant class imbalance in our data, we employed Borderline-SMOTE technique within our cross-validation framework. The results showed that the EEG-based models consistently outperformed those based on demographic data, particularly on balanced metrics such as MCC and F1-score that are especially crucial for evaluating performance on imbalanced data [46]. This superiority strongly supports that EEG captures a unique and powerful neurophysiological signal of amyloid pathology that is not present in standard clinical variables.

While the demographic model at baseline retained a marginally higher specificity, this was offset by a significantly lower sensitivity. In contrast, the EEG-based model demonstrated a much more balanced performance, showing a superior ability to correctly identify the challenging, clinically crucial, and minority A + SCD. This balanced strength is reflected in its higher MCC and F1-score, identifying the EEG-based models as the more reliable and effective classifier overall.

Taken together, these results support the feasibility of EEG, particularly when combined with machine learning, as a non-invasive, scalable, and sensitive approach for early AD diagnosis and risk stratification in SCD populations. Its potential application in routine screening or trial enrichment strategies could improve the identification of preclinical AD cases prior to the onset of clinical symptoms.

Lastly, in our study, the A-SCD demonstrated modest but statistically significant improvements in several cognitive tests over the study period. This pattern is most plausibly explained by practice effect from repeated testing, a well-documented phenomenon in longitudinal studies of cognitively unimpaired individuals. Such improvements should therefore not be interpreted as true cognitive gains. The presence of these expected learning gains in the A-SCD underscores their relative cognitive stability, despite their subjective complaints. In contrast, the A + SCD failed to show a similar phenomenon, exhibiting declining instead. Additionally, as a recent study suggests, this absence of practice effects in individuals with SCD may be a subtle but clinically informative marker, as it has been associated with a higher risk of progression to MCI [47].

### Limitations and future directions

This study had several limitations. First, although this study included 106 participants, only 24 were amyloid-positive. This limits the statistical power and may also influence the classification model evaluation, as our procedure did not employ nested cross-validation, meaning the reported classification performances might be interpreted as potentially optimistic. Second, the significance of EEG features from our pattern-generating statistical tests should be considered preliminary and interpreted with caution, as the analyses were not adjusted for multiple comparisons. Third, our analysis was restricted to the 1–55 Hz frequency range to avoid potential artifacts and the technical constraints imposed by our sampling rate. Future studies are necessary to investigate potential alterations in high gamma range (> 60 Hz). Finally, the hospital-based recruitment and 2-year follow-up period may not fully capture the long-term trajectory of the general population. Larger, population-based, and longer-duration cohort studies are warranted to validate these findings and to establish the prognostic value of EEG for predicting progression to MCI or AD.

Despite these limitations, this study demonstrated the feasibility and clinical potential of EEG as a sensitive biomarker for early AD-related changes in individuals with SCD. EEG offers several advantages over traditional biomarkers; it is non-invasive, cost-effective, and amenable to repeated measurements. Moreover, the combination of EEG with machine learning provides a powerful platform for the early detection, risk stratification, and monitoring of disease progression or treatment response. Future studies should focus on integrating multimodal EEG markers and employing deep learning methods to enhance the predictive accuracy and clinical applicability.

## Conclusions

This longitudinal multicenter study demonstrates that resting-state EEG can sensitively capture early and evolving functional brain alterations linked to amyloid pathology in subjective cognitive decline. Beyond confirming the classical phenomenon of ‘EEG slowing’, we identified a temporal shift from delta to theta elevation, persistent alpha network disruption consistent with an early disconnection syndrome, and the unexpected early emergence of gamma abnormalities, likely reflecting the vulnerability of GABAergic interneurons to amyloid toxicity. Importantly, these findings remained robust after addressing key confounders, including class imbalance and APOE4 status, through rigorous statistical controls and sensitivity analyses. Machine learning models using spectral and network EEG features consistently outperformed demographic models, and SHAP-based interpretation highlighted distinct electrophysiological signatures of amyloid burden. Furthermore, cognitive trajectories underscored that the absence of practice effects in amyloid-positive individuals may represent an additional subtle clinical marker at the preclinical stage.

Taken together, our results extend the current understanding of preclinical Alzheimer’s disease by revealing novel electrophysiological markers—such as early gamma dysfunction and progressive oscillatory slowing—and by demonstrating the interpretability and robustness of EEG-based machine learning. Given its accessibility, scalability, and sensitivity, EEG holds promise as a practical biomarker for individualized risk stratification, longitudinal monitoring, and trial enrichment in the earliest phases of the Alzheimer’s disease continuum.

## Supplementary Information

Below is the link to the electronic supplementary material.

Supplementary Material 1

## Data Availability

The data supporting these findings are available from the corresponding author upon reasonable request.

## Abbreviations

Abbreviation: Full Term
SCD: Subjective Cognitive Decline
AD: Alzheimer’s Disease
MCI: Mild Cognitive Impairment
PET: Positron Emission Tomography
CSF: Cerebrospinal Fluid
MRI: Magnetic Resonance Imaging
EEG: Electroencephalography
A + SCD: Amyloid-Positive Subjective Cognitive Decline
A–SCD: Amyloid-Negative Subjective Cognitive Decline
APOE4: Apolipoprotein Epsilon 4
SNSB: Seoul Neuropsychological Screening Battery
CDR: Clinical Dementia Rating
SVLT: Seoul Verbal Learning Test
K-MMSE: Korean Version of the Mini-Mental State Examination
DST-F: Digit Span Test–Forward
K-BNT: Korean Version of the Boston Naming Test
RCFT-C: Rey Complex Figure Test–Copy
RCFT-DR: Rey Complex Figure Test–Delayed Recall
DSC: Digit Symbol Coding
COWAT: Controlled Oral Word Association Test
K-TMT-B: Korean Trail Making Test B
K-CWST: Korean Color Word Stroop Test
SUVRs: Standardized Uptake Value Ratios
MCC: Matthews Correlation Coefficient
SHAP: Shapley Additive exPlanations
