# Supplementary material for: EEG-based detection of early functional brain changes in subjective cognitive decline: a prospective cohort study
Source: Alzheimers Res Ther. 2025 Dec 29;17:265. doi: 10.1186/s13195-025-01875-8 (PMC12751729; doi:10.1186/s13195-025-01875-8)

**Supplementary Paragraph 1.**

**EEG recording and preprocessing**

The participants underwent EEG recordings in a resting state for at least 3 minutes while closing their eyes. EEG recordings were obtained at baseline and follow-up. A total of 19 electrodes were attached according to the international 10-20 system: Fp1, Fp2, F7, F3, Fz, F4, F8, T3, C3, Cz, C4, T4, T5, P3, Pz, P4, T6, O1, and O2. Notch filter was applied at 60 Hz to remove powerline noise. The reference electrode was linked ear electrode (average of A1 and A2). Electrode impedance was kept below 10 kΩ, and the sampling rate was 200, 250, or 400 Hz.

All preprocessing of EEG data was performed using MATLAB R2021b (MathWorks, Natick, MA, USA) and the EEGLAB toolbox. Before preprocessing, to match the sampling rate of each EEG data, all EEG data were downsampled to 200 Hz. This approach was chosen over up-sampling to preserve the reliability of the original signals. The EEG data from each participant underwent baseline correction to eliminate the DC drift. A finite impulse response (FIR) filter ranging from 1 to 55 Hz was adopted, avoiding potential signal distortion or information loss caused by attenuated 60 Hz. To remove the sinusoidal noise at 60 Hz and their harmonic noise, the CleanLine plug-in was used. All the EEG data were visually inspected to determine an electrode to be interpolated. The selected bad electrode was interpolated using spherical interpolation in the EEGLAB. Artifact subspace reconstruction (ASR) was performed to calibrate abnormally large amplitude artifacts. The common average reference (CAR) and reference electrode standardization technique (REST) were used for re-referencing. Independent component analysis (ICA) was conducted to eliminate any possible noise. We used the ICLabel toolbox to classify the independent components into the following categories based on a neural network trained on crowdsourced data, with the probability of being in each category: (1) brain, (2) muscle, (3) eye, (4) heart, (5) line noise, (6) channel noise, and (7) others. Independent components with a probability of being classified as noise of >70% were removed. All continuous EEG data were segmented into 2-s epochs, and an epoch at any of the 19 channels with any amplitude over ± 75 μV was excluded due to the possibility of containing significant physiological artifacts. Finally, to ensure a balanced and unbiased comparison, 60 epochs, the minimum number of epochs available from any single participant, were randomly selected for each participant to ensure a consistent number.

**Power spectral analysis**

The absolute spectral power was computed for each 2-s epoch using Welch’s method with a Hamming window. The absolute spectral power calculated for each 2-s epoch was separated into the following frequency bands and summed: delta (1-4 Hz), theta (4-8 Hz), alpha (8-12 Hz), beta (12-30 Hz), and gamma (30-55 Hz). The frequency bin was set to 0.5 Hz. Based on the absolute spectral power, the relative spectral power was calculated by normalizing the absolute spectral power in each frequency band with the absolute spectral power in the full band (1–55 Hz). Subsequently, the relative spectral power was included in the further analysis.

**Network analysis**

The preprocessed EEG data were filtered into the same frequency band in the spectral power analysis using an FIR bandpass filter. To construct a graph-theory-based weighted network, electrodes and functional connectivity between each pair of electrodes were used as nodes and edges in the network, respectively. For the functional connectivity, the weighted phase lag index (wPLI) ranging from 0 to 1 was estimated for each pair of electrodes in each frequency band. To consider possible spurious connections in the network, we adopted proportional thresholding in the network analysis. The thresholding proportion was set from 5% to 35% in increments of 5%, and for each thresholding proportion, weaker connections within the proportion were removed. In each network with weak connections removed according to the thresholding proportion, the following network indices were determined: clustering coefficient (CC), node strength (NS), and efficiency. Finally, the network indices for each threshold proportion were averaged. In this study, the CC and NS were computed at the global and nodal levels, and efficiency was assessed only at the global level. Network analysis was performed using the Brain Connectivity toolbox.

**Supplementary Table 1.**

Cognitive trajectory of study participants.

| Cognitive test,  group | Baseline | Follow-up | Cohen’s d  (95% CI) | *P* |
| --- | --- | --- | --- | --- |
| K-MMSE |  |  |  |  |
| A+SCD | 26.88 ± 2.37 | 26.42 ± 2.78 | 0.20 | .328 |
| A-SCD | 27.28 ± 1.85 | 28.10 ± 1.75 | -0.40 | **.001** |
| K-BNT |  |  |  |  |
| A+SCD | 54.02 ± 30.71 | 52.91 ± 36.56 | 0.04 | .842 |
| A-SCD | 59.71 ± 26.28 | 68.32 ± 25.88 | -0.42 | **< .001** |
| RCFT-DR |  |  |  |  |
| A+SCD | 43.88 ± 26.44 | 48.67 ± 32.65 | -0.20 | .328 |
| A-SCD | 47.79 ± 23.80 | 59.30 ± 27.40 | -0.44 | **< .001** |
| SVLT |  |  |  |  |
| A+SCD | 23.13 ± 15.10 | 30.69 ± 26.00 | -0.34 | .107 |
| A-SCD | 28.02 ± 13.06 | 52.21 ± 24.91 | -1.09 | **< .001** |
| DSC |  |  |  |  |
| A+SCD | 52.82 ± 33.17 | 57.83 ± 32.43 | -0.18 | .397 |
| A-SCD | 64.04 ± 24.06 | 69.56 ± 24.89 | -0.29 | **.012** |
| K-TMT-B |  |  |  |  |
| A+SCD | 58.42 ± 22.86 | 47.33 ± 25.90 | 0.51 | **.020** |
| A-SCD | 63.86 ± 21.10 | 67.73 ± 21.02 | -0.19 | .084 |

Bold indicates a significant *P*-value (*P* < .05); A+SCD, amyloid-positive subjective cognitive decline; A–SCD, amyloid-negative subjective cognitive decline; K-MMSE, Korean version of the Mini-Mental State Examination; K-BNT, Korean version of the Boston Naming Test; RCFT-DR, Rey Complex Figure Test–Delayed Recall; SVLT, Seoul Verbal Learning Test; DSC, Digit Symbol Coding; K-TMT-B, Korean version of Trail Making Test B.

**Supplementary Table 2.**

Significant correlations between EEG features and global SUVR and cognitive tests at baseline.

| Measures | Frequency band | EEG feature | Partial correlation  coefficient | *P* |
| --- | --- | --- | --- | --- |
| Global SUVR | Delta | T5 | 0.206 | **.035** |
|  |  | O1 | 0.266 | **.006** |
|  |  | T3 (CC) | 0.205 | **.036** |
|  |  | T4 (NS) | 0.196 | **.045** |
|  | Theta | O1 | 0.277 | **.004** |
|  | Alpha | O1 | -0.288 | **.019** |
|  |  | O2 | -0.215 | **.027** |
|  | Beta | T3 | -0.215 | **.027** |
| K-MMSE | Delta | F7 | -0.239 | **.014** |
|  |  | Fz | -0.202 | **.039** |
|  |  | Pz | -0.208 | **.033** |
|  | Alpha | Fp1 (CC) | 0.313 | **.001** |
|  |  | F7 (CC) | 0.294 | **.002** |
|  |  | F3 (CC) | 0.286 | **.003** |
|  |  | Fz (CC) | 0.310 | **.001** |
|  |  | F4 (CC) | 0.290 | **.003** |
|  |  | Cz (CC) | 0.294 | **.002** |
|  |  | C4 (CC) | 0.273 | **.005** |
|  |  | T5 (CC) | 0.287 | **.003** |
|  |  | Pz (CC) | 0.319 | **.001** |
|  |  | Global (CC) | 0.299 | **.002** |
|  |  | C4 (NS) | 0.253 | **.009** |
|  |  | Pz (NS) | 0.251 | **.010** |
|  |  | O2 (NS) | 0.310 | **.001** |
| DST-F | Delta | F7 | -0.207 | **.034** |
|  | Theta | O1 | -0.203 | **.037** |
|  | Beta | T3 | 0.195 | **.046** |
| K-BNT | Delta | F4 | -0.219 | **.025** |
|  | Alpha | Fp1 (CC) | 0.242 | **.013** |
|  |  | F7 (CC) | 0.264 | **.007** |
|  |  | F3 (CC) | 0.241 | **.013** |
|  |  | Fz (CC) | 0.249 | **.010** |
|  |  | F4 (CC) | 0.234 | **.016** |
|  |  | Cz (CC) | 0.257 | **.008** |
|  |  | C4 (CC) | 0.235 | **.016** |
|  |  | T5 (CC) | 0.238 | **.014** |
|  |  | Pz (CC) | 0.220 | **.024** |
|  |  | Global (CC) | 0.244 | **.012** |
|  |  | O2 (NS) | 0.222 | **.023** |
| SVLT | Theta | O1 | -0.243 | **.012** |
|  | Beta | T3 | 0.206 | **.035** |
| DSC | Theta | O1 | -0.246 | **.011** |
| COWAT | Alpha | Fp1 (CC) | 0.229 | **.019** |
|  |  | F7 (CC) | 0235 | **.016** |
|  |  | F3 (CC) | 0.228 | **.019** |
|  |  | Fz (CC) | 0.246 | **.011** |
|  |  | F4 (CC) | 0.227 | **.020** |
|  |  | Cz (CC) | 0.253 | **.009** |
|  |  | C4 (CC) | 0.239 | **.014** |
|  |  | T5 (CC) | 0.218 | **.025** |
|  |  | Pz (CC) | 0.245 | **.012** |
|  |  | Global (CC) | 0.227 | **.020** |
|  |  | C4 (NS) | 0.210 | **.031** |
|  |  | O2 (NS) | 0.246 | **.011** |
| K-CWST | Delta | Pz | -0.290 | **.003** |
|  |  | T3 (CC) | -0.237 | **.015** |
|  | Theta | O1 | -0.211 | **.030** |

Bold indicates a significant *P*-value (*P* < .05); SUVR, standardized uptake value ratio; K-MMSE, Korean version of the Mini-Mental State Examination; DST-F, Digit Span Test: Forward; K-BNT, Korean version of the Boston Naming Test; SVLT, Seoul Verbal Learning Test; DSC, Digit Symbol Coding; COWAT, Controlled Oral Word Association Test; K-CWST, Korean version of the Color Word Stroop Test; CC, clustering coefficient; NS, node strength.

**Supplementary Table 3.**

Significant correlations between EEG features and both global SUVR and cognitive tests at follow-up.

| Measures | Frequency band | EEG feature | Partial correlation  coefficient | *P* |
| --- | --- | --- | --- | --- |
| Global SUVR | Theta | F3 | 0.214 | **.029** |
|  |  | Fz | 0.285 | **.003** |
|  |  | T5 | 0.337 | **< .001** |
|  |  | O1 | 0.342 | **< .001** |
|  |  | O2 | 0.319 | **.001** |
|  | Gamma | Fp1 (CC) | -0.281 | **.004** |
|  |  | T6 (CC) | -0.294 | **.002** |
|  |  | Fz (NS) | -0.318 | **.001** |
|  |  | P4 (NS) | -0.205 | **.036** |
| K-MMSE | Alpha | F3 (CC) | 0.192 | **.050** |
|  |  | C3 (CC) | 0.218 | **.025** |
|  |  | T5 (NS) | 0.205 | **.035** |
| DST-F | Theta | F4 | -0.211 | **.031** |
|  |  | T3 | -0.268 | **.006** |
|  |  | C4 | -0.229 | **.019** |
|  |  | T4 | -0.308 | **.001** |
|  |  | T5 | -0.292 | **.003** |
|  |  | P3 | -0.256 | **.008** |
|  |  | Pz | -0.236 | **.016** |
|  |  | T6 | -0.262 | **.007** |
|  |  | O1 | -0.253 | **.009** |
|  |  | O2 | -0.236 | **.016** |
|  |  | Global | -0.241 | **.013** |
| K-BNT | Theta | T3 | -0.250 | **.010** |
|  |  | T5 | -0.286 | **.003** |
|  |  | P3 | -0.269 | **.006** |
|  |  | O1 | -0.280 | **.004** |
|  | Alpha | C3 (CC) | 0.227 | **.020** |
|  |  | T5 (NS) | 0.226 | **.020** |
|  | Gamma | Fp1 (CC) | 0.212 | **.030** |
| RCFT-DR | Alpha | F3 (CC) | 0.202 | **.039** |
|  |  | P4 (NS) | 0.198 | **.043** |
| DSC | Theta | Fp1 | -0.230 | **.018** |
|  |  | F3 | -0.196 | **.045** |
|  |  | F4 | -0.238 | **.014** |
|  |  | C3 | -0.229 | **.019** |
|  |  | Cz | -0.282 | **.004** |
|  |  | C4 | -0.272 | **.005** |
|  |  | T4 | -0.291 | **.003** |
|  |  | T5 | -0.281 | **.004** |
|  |  | T6 | -0.278 | **.004** |
|  |  | P3 | -0.327 | **.001** |
|  |  | Pz | -0.310 | **.001** |
|  |  | P4 | -0.271 | **.005** |
|  |  | O1 | -0.256 | **.008** |
|  |  | O2 | -0.261 | **.007** |
|  |  | Global | -0.285 | **.003** |
| COWAT | Theta | Fp1 | -0.220 | **.024** |
|  |  | C3 | -0.200 | **.041** |
|  |  | Cz | -0.244 | **.012** |
|  |  | C4 | -0.204 | **.037** |
|  |  | T5 | -0.197 | **.044** |
|  |  | P3 | -0.216 | **.027** |
|  |  | Pz | -0.252 | **.010** |
|  |  | P4 | -0.203 | **.038** |
|  |  | O1 | -0.231 | **.018** |
|  |  | Global | -0.215 | **.028** |
|  | Alpha | C3 (CC) | 0.227 | **.020** |
|  |  | P4 (NS) | 0.201 | **.040** |
| K-TMT-B | Theta | Pz | -0.211 | **.031** |
| K-CWST | Alpha | Fp2 (NS) | 0.241 | **.013** |

Bold indicates a significant *P*-value (*P* < .05); SUVR, standardized uptake value ratio; K-MMSE, Korean version of the Mini-Mental State Examination; DST-F, Digit Span Test: Forward; K-BNT, Korean version of the Boston Naming Test; RCFT-DR, Rey Complex Figure Test–Delayed Recall; DSC, Digit Symbol Coding; COWAT, Controlled Oral Word Association Test; K-TMT-B, Korean version of Trail Making Test B; K-CWST, Korean version of the Color Word Stroop Test; CC, clustering coefficient; NS, node strength.

**Supplementary Table 4.**

Mean and standard deviation of selected features at baseline.

| Feature | A+SCD  (*N* = 24) | A–SCD  (*N* = 82) | *P* |
| --- | --- | --- | --- |
| **Baseline (EEG)** |  |  |  |
| C4 delta (CC) | 0.27 ± 0.02 | 0.26 ± 0.02 | .821 |
| Global delta (CC) | 0.27 ± 0.02 | 0.27 ± 0.01 | .169 |
| T3 delta (NS) | 4.62 ± 0.68 | 4.65 ± 0.80 | .948 |
| C4 alpha (CC) | 0.27 ± 0.07 | 0.32 ± 0.09 | **.017** |
| P4 alpha (CC) | 0.28 ± 0.07 | 0.32 ± 0.10 | .059 |
| P4 alpha (NS) | 5.24 ± 1.56 | 6.18 ± 2.22 | .062 |
| T5 beta (CC) | 0.14 ± 0.03 | 0.14 ± 0.03 | .987 |
| O2 beta (CC) | 0.14 ± 0.04 | 0.15 ± 0.03 | .926 |
| Cz beta (NS) | 2.36 ± 1.04 | 2.54 ± 0.85 | .636 |
| Cz gamma | 3.82 ± 2.24 | 4.32 ± 3.61 | .666 |
| C4 gamma | 6.41 ± 6.95 | 7.03 ± 7.64 | .722 |
| Fp1 gamma (CC) | 0.10 ± 0.01 | 0.11 ± 0.02 | .086 |
| T4 gamma (CC) | 0.11 ± 0.01 | 0.11 ± 0.02 | .615 |
| Global gamma (CC) | 0.11 ± 0.01 | 0.11 ± 0.01 | .325 |
| T3 gamma (NS) | 1.61 ± 0.26 | 1.67 ± 0.41 | .603 |
| **Baseline (Demographic)** |  |  |  |
| APOE4 carrier (n, %) | 12 (50.0) | 9 (11.0) | **< .001** |
| K-MMSE | 26.88 ± 2.36 | 27.28 ± 1.85 | .379 |
| DSC | 52.82 ± 33.17 | 64.04 ± 24.06 | .070 |
| Sex (male, %) | 14 (58.3) | 32 (39.0) | .093 |
| Framingham  cardiovascular risk score | 10.98 ± 8.78 | 8.39 ± 7.06 | .139 |
| Age | 73.42 ± 5.64 | 69.82 ± 6.05 | **.011** |
| RCFT-C | 60.83 ± 21.73 | 57.00 ± 22.24 | .458 |
| Body fat (%) | 26.10 ± 7.71 | 30.12 ± 7.90 | **.030** |
| Education (years) | 12.71 ± 4.07 | 11.04 ± 3.88 | .069 |

Bold indicates a significant *P*-value (*P* < .05); A+SCD, amyloid-positive subjective cognitive decline; A–SCD, amyloid-negative subjective cognitive decline; CC, clustering coefficient; NS, node strength; APOE4, apolipoprotein epsilon 4; K-MMSE, Korean version of the Mini-Mental State Examination; DSC, Digit Symbol Coding; RCFT-C, Rey Complex Figure Test–Copy.

**Supplementary Table 5.**

Mean and standard deviation of selected features at follow-up.

| Feature | A+SCD  (*N* = 24) | A–SCD  (*N* = 82) | *P* |
| --- | --- | --- | --- |
| **Follow-up (EEG)** |  |  |  |
| Fz delta (NS) | 4.09 ± 0.84 | 4.42 ± 0.76 | .114 |
| T6 delta (NS) | 4.66 ± 0.61 | 4.44 ± 0.76 | .161 |
| F7 theta (NS) | 3.43 ± 0.67 | 3.67 ± 0.93 | .265 |
| F4 theta (NS) | 4.07 ± 0.85 | 3.92 ± 1.00 | .831 |
| Fp1 alpha | 34.50 ± 20.12 | 33.11 ± 18.64 | .631 |
| Global alpha (CC) | 0.28 ± 0.06 | 0.31 ± 0.08 | .067 |
| T3 alpha (NS) | 3.93 ± 1.06 | 4.06 ± 1.60 | .704 |
| Fp1 beta (CC) | 0.13 ± 0.02 | 0.14 ± 0.04 | .672 |
| F7 beta (CC) | 0.13 ± 0.02 | 0.14 ± 0.03 | .663 |
| C4 beta (CC) | 0.13 ± 0.03 | 0.14 ± 0.04 | .573 |
| P3 beta (CC) | 0.13 ± 0.03 | 0.14 ± 0.04 | .539 |
| O2 beta (NS) | 2.28 ± 0.63 | 2.69 ± 0.87 | .133 |
| T6 gamma (CC) | 0.10 ± 0.01 | 0.11 ± 0.01 | **.016** |
| **Follow-up (Demographic)** |  |  |  |
| APOE4 carrier (n, %) | 12 (50.0) | 9 (11.0) | **< .001** |
| RCFT-DR | 48.67 ± 32.65 | 59.30 ± 27.40 | .113 |
| Age | 75.42 ± 5.64 | 71.82 ± 6.05 | **.011** |
| Body fat (%) | 27.74 ± 7.35 | 31.91 ± 8.01 | **.024** |
| Framingham  cardiovascular risk score | 10.13 ± 7.73 | 8.36 ± 6.50 | .265 |
| K-ECOG | 76.50 ± 29.58 | 72.57 ± 22.45 | .486 |
| DSC | 57.83 ± 32.43 | 69.56 ± 24.89 | .061 |
| Education (years) | 12.71 ± 4.07 | 11.04 ± 3.88 | .069 |
| K-MMSE | 26.42 ± 2.78 | 28.10 ± 1.75 | **.001** |

Bold indicates a significant *P*-value (*P* < .05); A+SCD, amyloid-positive subjective cognitive decline; A–SCD, amyloid-negative subjective cognitive decline; CC, clustering coefficient; APOE4, apolipoprotein epsilon 4; RCFT-DR, Rey Complex Figure Test–Delayed Recall; K-ECOG, Korean version of Everyday Cognition; DSC, Digit Symbol Coding; K-MMSE, Korean version of the Mini-Mental State Examination.

**Supplementary Table 6.**

Baseline demographic characteristics of the study participants according to APOE4 status.

|  | APOE4 carrier  (*N* = 21) | APOE4 non-carrier  (*N* = 85) | *P* |
| --- | --- | --- | --- |
| Age (years) | 71.19 ± 7.13 | 70.49 ± 5.89 | .643 |
| Sex, male (n, %) | 8 (38.1) | 38 (44.7) | .584 |
| Education (years) | 11.38 ± 4.13 | 11.42 ± 3.95 | .965 |
| Global SUVR | 1.48 ± 0.34 | 1.24 ± 0.19 | **< .001** |
| BMI | 24.92 ± 3.30 | 24.83 ± 3.21 | .904 |
| Body fat (%) | 29.68 ± 8.56 | 29.09 ± 7.90 | .765 |
| Body muscle (%) | 24.43 ± 4.82 | 24.35 ± 6.62 | .957 |
| Visceral fat (%) | 8.67 ± 3.69 | 9.24 ± 3.83 | .541 |
| Waist circumference (cm) | 86.97 ± 9.81 | 86.29 ± 9.40 | .772 |
| Framingham cardiovascular risk score | 9.14 ± 7.49 | 8.94 ± 7.56 | .913 |
| K-ECOG | 64.62 ± 20.68 | 71.68 ± 21.82 | .183 |
| Periventricular WMH (grade 1/2/3) | 15/4/2 | 56/16/13 | .789 |
| Deep WMH (grade 1/2/3) | 18/2/1 | 61/19/5 | .391 |
| Lacune (n, %) | 1 (4.8) | 7 (8.2) | .589 |
| Cerebral microbleed (n, %) | 1 (4.8) | 7 (8.2) | .589 |

Bold indicates a significant P-value (P < .05); APOE4, apolipoprotein epsilon 4; SUVR, standardized uptake value ratio; BMI, body mass index; K-ECOG, Korean version of Everyday Cognition; WMH, white-matter hyperintensity.

**Supplementary Table 7.**

Cognitive tests between the APOE4 carrier and APOE4 non-carrier at baseline and follow-up.

|  | APOE4 carrier  (*N* = 21) | APOE4 non-carrier  (*N* = 85) | Cohen’s d  (95% CI) | *P* |
| --- | --- | --- | --- | --- |
| **Baseline** | ± |  |  |  |
| K-MMSE | 27.14 ± 2.29 | 27.20 ± 1.91 | 0.03 (-0.45, 0.51) | .906 |
| DST-F | 52.32 ± 27.03 | 64.66 ± 30.45 | 0.41 (-0.07, 0.89) | .092 |
| K-BNT | 55.83 ± 26.50 | 59.06 ± 27.60 | 0.12 (-0.36, 0.60) | .630 |
| RCFT-C | 59.96 ± 22.07 | 57.35 ± 22.18 | -0.12 (-0.60, 0.36) | .630 |
| RCFT-DR | 54.13 ± 25.71 | 45.12 ± 23.82 | -0.37 (-0.85, 0.11) | .129 |
| SVLT | 23.38 ± 14.88 | 27.78 ± 13.25 | 0.32 (-0.16, 0.80) | .186 |
| DSC | 57.72 ± 31.42 | 62.43 ± 25.45 | 0.18 (-0.30, 0.65) | .471 |
| COWAT | 55.70 ± 30.44 | 52.32 ± 28.11 | -0.12 (-0.60, 0.36) | .628 |
| K-TMT-B | 67.05 ± 20.89 | 61.53 ± 21.66 | -0.25 (-0.74, 0.22) | .295 |
| K-CWST | 31.28 ± 29.46 | 54.23 ± 25.06 | -0.27 (-0.75, 0.21) | .267 |
| **Follow-up** |  |  |  |  |
| K-MMSE | 26.86 ± 2.97 | 27.93 ± 1.83 | 0.51 (0.30, 0.99) | **.038** |
| DST-F | 58.28 ± 30.17 | 69.08 ± 28.42 | 0.38 (-0.11, 0.86) | .126 |
| K-BNT | 57.1 ± 337.51 | 66.73 ± 26.66 | 0.33 (-0.15,0.81) | .178 |
| RCFT-C | 56.00 ± 25.58 | 51.69 ± 25.00 | -0.17 (-0.65, 0.31) | .483 |
| RCFT-DR | 53.48 ± 32.48 | 57.74 ± 28.04 | 0.15 (-0.33, 0.63) | .547 |
| SVLT | 39.17 ± 29.61 | 49.36 ± 25.61 | 0.39 (-0.10, 0.87) | .117 |
| DSC | 60.36 ± 32.04 | 58.52 ± 25.65 | 0.30 (-0.18, 0.78) | .218 |
| COWAT | 51.81 ± 29.69 | 57.61 ± 29.26 | 0.20 (-0.28, 0.68) | .419 |
| K-TMT-B | 59.87 ± 25.90 | 63.91 ± 23.21 | 0.17 (-0.31, 0.65) | .487 |
| K-CWST | 59.42 ± 29.17 | 59.14 ± 28.63 | -0.10 (-0.48, 0.47) | .968 |

Bold indicates a significant P-value (P < .05); APOE4, apolipoprotein epsilon 4; CI, confidence interval; K-MMSE, Korean version of the Mini-Mental State Examination; DST-F, Digit Span Test: Forward; K-BNT, Korean version of the Boston Naming Test; RCFT-C, Rey Complex Figure Test–Copy; RCFT-DR, Rey Complex Figure Test–Delayed Recall; SVLT, Seoul Verbal Learning Test; DSC, Digit Symbol Coding; COWAT, Controlled Oral Word Association Test; K-TMT-B, Korean version of the Trail Making Test B; K-CWST, Korean version of the Color Word Stroop Test.

**Supplementary Table 8.**

EEG spectral and network features showing significant differences between the APOE4 carrier and APOE4 non-carrier at baseline and follow-up.

| Phase | Frequency  band | Feature | APOE4  carrier  (*N* = 21) | APOE4  non-carrier  (*N* = 85) | Effect size  (Partial η^2^) | *P* |
| --- | --- | --- | --- | --- | --- | --- |
| Baseline | Delta | F7 | 42.17 ± 15.74 | 33.35 ± 13.48 | 0.04 | **0.050** |
|  | Theta | C3 (NS) | 3.45 ± 0.81 | 3.89 ± 1.06 | 0.04 | **0.040** |
|  | Alpha | F7 (NS) | 5.38 ± 2.27 | 4.62 ± 1.69 | 0.05 | **0.027** |
|  | Beta | Fz (CC) | 0.15 ± 0.05 | 0.14 ± 0.03 | 0.04 | **0.036** |
|  |  | F4 (CC) | 0.15 ± 0.04 | 0.14 ± 0.03 | 0.04 | **0.048** |
|  |  | Cz (CC) | 0.15 ± 0.05 | 0.14 ± 0.03 | 0.04 | **0.050** |
|  |  | C4 (CC) | 0.15 ± 0.06 | 0.14 ± 0.03 | 0.04 | **0.048** |
|  |  | C4 (NS) | 2.95 ± 1.27 | 2.54 ± 0.71 | 0.04 | **0.037** |
|  |  | T3 (CC) | 0.15 ± 0.04 | 0.13 ± 0.02 | 0.05 | **0.027** |
|  |  | P4 (NS) | 3.12 ± 1.29 | 2.72 ± 0.66 | 0.05 | **0.028** |
|  | Gamma | Fp2 (NS) | 1.93 ± 0.49 | 1.78 ± 0.42 | 0.04 | **0.049** |
| Follow-up | Delta | O2 | 4.18 ± 0.76 | 4.52 ± 0.86 | 0.04 | **0.038** |
|  | Theta | F4 | 19.94 ± 7.24 | 14.86 ± 7.42 | 0.04 | **0.039** |
|  |  | F7 (NS) | 3.23 ± 0.64 | 3.71 ± 0.91 | 0.05 | **0.024** |
|  | Beta | Fp1 (CC) | 0.15 ± 0.05 | 0.14 ± 0.03 | 0.06 | **0.009** |
|  |  | Fp2 (CC) | 0.15 ± 0.05 | 0.14 ± 0.03 | 0.06 | **0.015** |
|  |  | F7 (CC) | 0.15 ± 0.05 | 0.14 ± 0.02 | 0.06 | **0.010** |
|  |  | F7 (NS) | 2.41 ± 1.03 | 2.10 ± 0.47 | 0.05 | **0.022** |
|  |  | F3 (NS) | 2.47 ± 0.71 | 2.16 ± 0.54 | 0.07 | **0.005** |
|  |  | Fz (CC) | 0.15 ± 0.05 | 0.14 ± 0.03 | 0.06 | **0.013** |
|  |  | F4 (CC) | 0.15 ± 0.05 | 0.14 ± 0.03 | 0.05 | **0.017** |
|  |  | F4 (NS) | 2.39 ± 1.00 | 2.11 ± 0.54 | 0.06 | **0.016** |
|  |  | F8 | 15.07 ± 5.42 | 19.77 ± 8.49 | 0.04 | **0.041** |
|  |  | F8 (CC) | 0.15 ± 0.05 | 0.14 ± 0.03 | 0.05 | **0.029** |
|  |  | F8 (NS) | 2.44 ± 0.97 | 2.14 ± 0.51 | 0.05 | **0.018** |
|  |  | T3 (CC) | 0.14 ± 0.05 | 0.13 ± 0.02 | 0.05 | **0.030** |
|  |  | Cz (CC) | 0.15 ± 0.06 | 0.14 ± 0.03 | 0.05 | **0.025** |
|  |  | C4 (CC) | 0.16 ± 0.06 | 0.14 ± 0.03 | 0.09 | **0.002** |
|  |  | C4 (NS) | 2.78 ± 1.32 | 2.45 ± 0.60 | 0.04 | **0.031** |
|  |  | T5 (CC) | 0.15 ± 0.05 | 0.14 ± 0.02 | 0.05 | **0.021** |
|  |  | P3 (CC) | 0.15 ± 0.05 | 0.14 ± 0.03 | 0.04 | **0.043** |
|  |  | Pz (CC) | 0.15 ± 0.06 | 0.14 ± 0.03 | 0.06 | **0.015** |
|  |  | Pz (NS) | 3.18 ± 1.46 | 2.71 ± 0.66 | 0.07 | **0.007** |
|  |  | P4 (CC) | 0.15 ± 0.06 | 0.14 ± 0.03 | 0.07 | **0.009** |
|  |  | P4 (NS) | 3.18 ± 1.47 | 2.70 ± 0.67 | 0.07 | **0.006** |
|  |  | T6 (CC) | 0.15 ± 0.05 | 0.14 ± 0.03 | 0.05 | **0.021** |
|  |  | T6 (NS) | 2.67 ± 0.97 | 2.50 ± 0.52 | 0.04 | **0.039** |
|  |  | O1 (CC) | 0.15 ± 0.06 | 0.14 ± 0.03 | 0.04 | **0.034** |
|  |  | O1 (NS) | 2.86 ± 1.10 | 2.56 ± 0.64 | 0.06 | **0.014** |
|  |  | O2 (CC) | 0.16 ± 0.06 | 0.14 ± 0.03 | 0.06 | **0.012** |
|  |  | Global (CC) | 0.15 ± 0.05 | 0.14 ± 0.02 | 0.05 | **0.017** |
|  |  | Global (CC) | 2.61 ± 0.90 | 2.37 ± 0.43 | 0.06 | **0.014** |
|  |  | Global (E) | 0.16 ± 0.06 | 0.15 ± 0.03 | 0.06 | **0.015** |
|  | Gamma | F3 | 5.09 ± 3.07 | 8.24 ± 7.94 | 0.04 | **0.033** |
|  |  | F4 (NS) | 1.97 ± 0.45 | 1.86 ± 0.42 | 0.04 | **0.049** |
|  |  | T4 (NS) | 1.76 ± 0.38 | 1.56 ± 0.28 | 0.07 | **0.006** |

Bold indicates a significant P-value (P < .05); APOE4, apolipoprotein epsilon 4; CC, clustering coefficient; NS, node strength; E, efficieny.

**Supplementary Fig. 1.**

Correlations between the global SUVR and EEG activity at (A) baseline and (B) follow-up.

SUVR, standardized uptake value ratio.


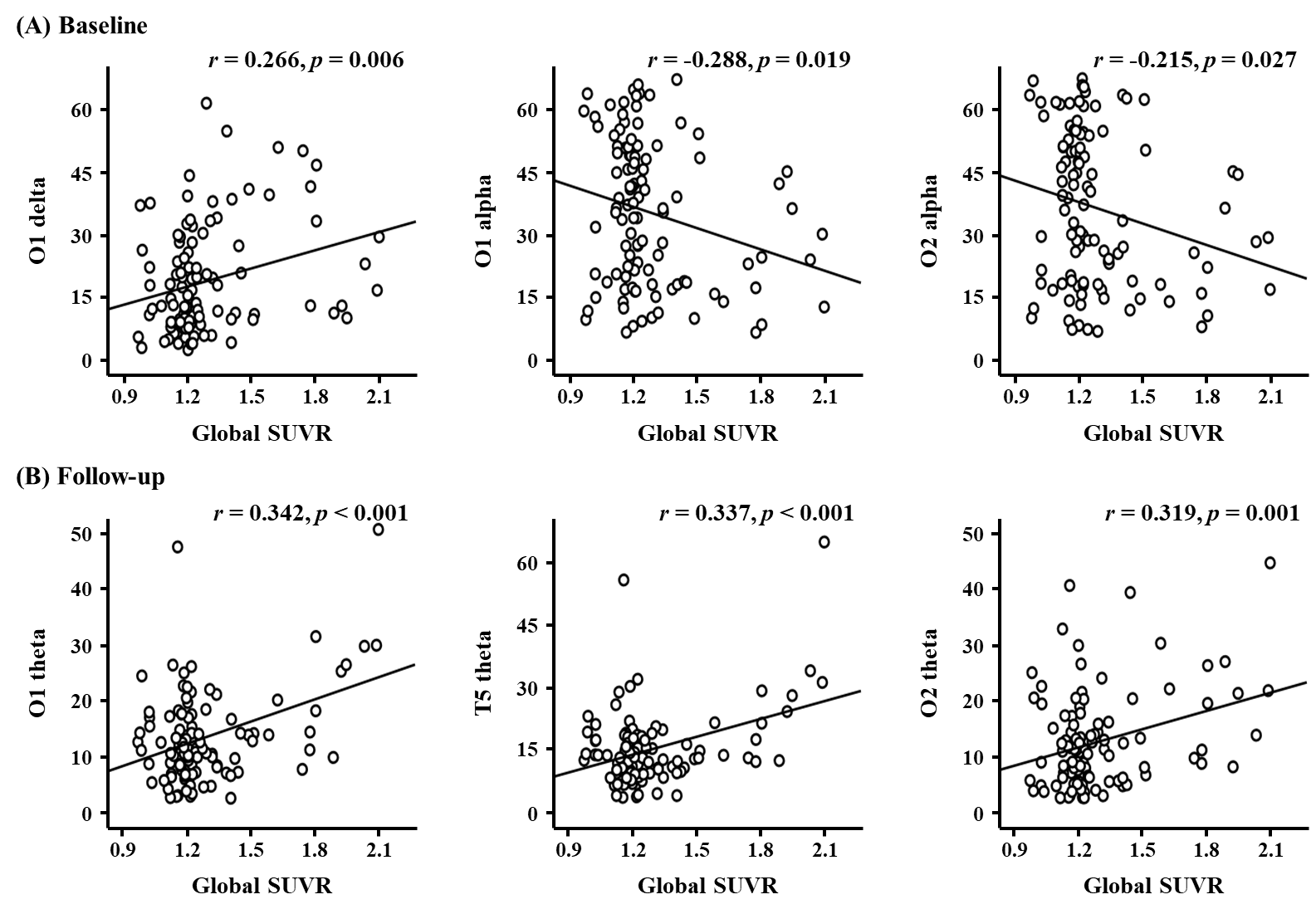


**Supplementary Fig. 2.**

Correlations between cognitive tests and EEG activity at (A) baseline and (B) follow-up.

K-MMSE, Korean version of the Mini-Mental State Examination; CC, clustering coefficient.


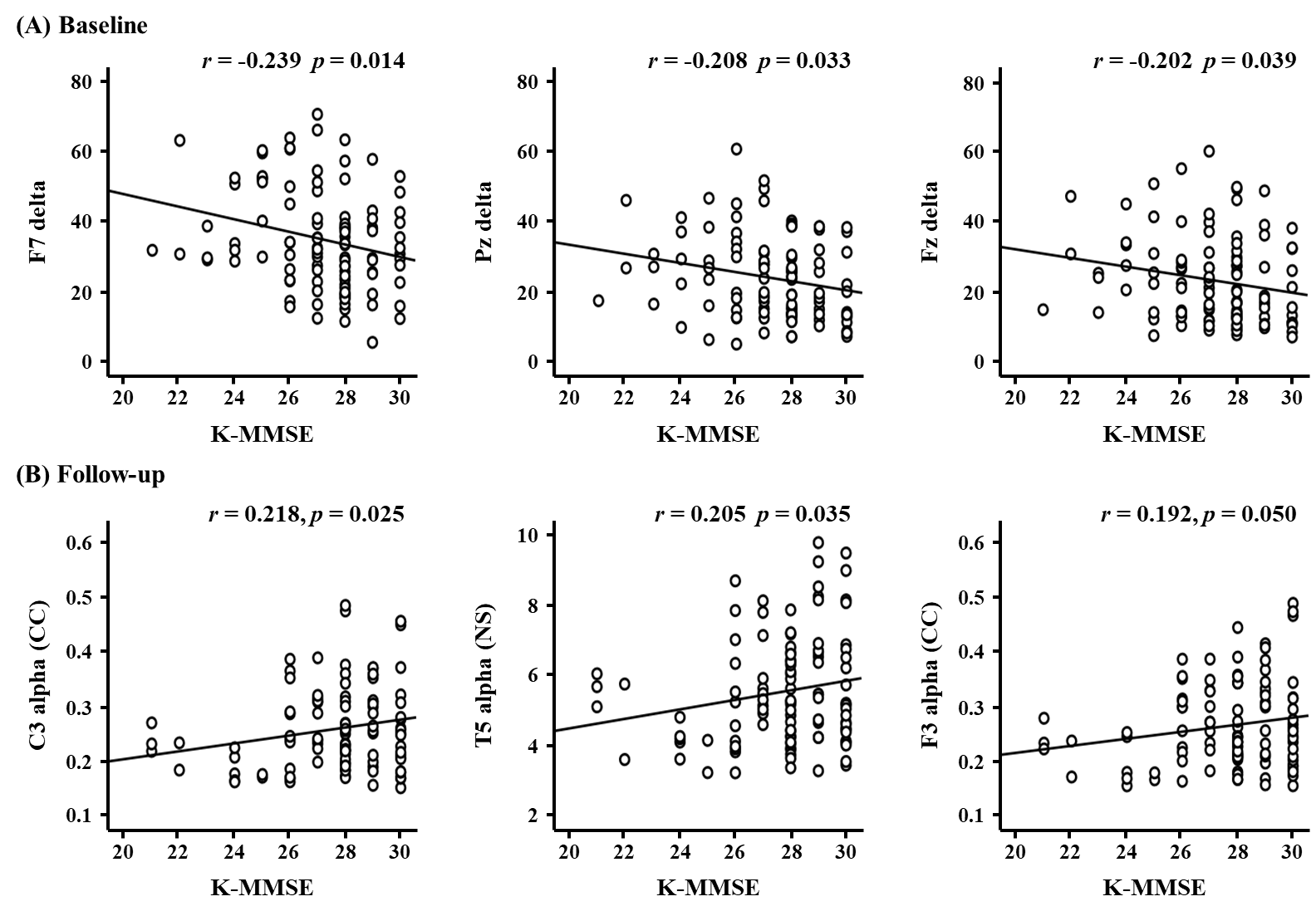

Supplement: Supplementary file 1 — Supplementary Material 1 [file 13195_2025_1875_MOESM1_ESM.doc]
